# Detection of *Cronobacter sakazakii* in powdered infant formula using an immunoliposome-based immunomagnetic concentration and separation assay

**DOI:** 10.1038/srep34721

**Published:** 2016-10-10

**Authors:** Shruti Shukla, Gibaek Lee, Xinjie Song, Jung Hyun Park, Hyunjeong Cho, Eun Ju Lee, Myunghee Kim

**Affiliations:** 1 Department of Food Science and Technology, Yeungnam University, 280 Daehak-ro, Gyeongsan-si, Gyeongsangbuk-do 38541, Republic of Korea; 2 Experiment and Research Institute, National Agricultural Products Quality Management Service, Gimcheon-si 39660, Republic of Korea; 3 Department of Medical Biotechnology, Yeungnam University, 280 Daehak-ro, Gyeongsan-si, Gyeongsangbuk-do 38541, Republic of Korea

## Abstract

This study aimed to optimize the applicability of an immunoliposome-based immunomagnetic concentration and separation assay to facilitate rapid detection of *Cronobacter sakazakii* in powdered infant formula (PIF). To determine the detection limit, specificity, and pre-enrichment incubation time (0, 4, 6, and 8 h), assay tests were performed with different cell numbers of *C. sakazakii* (2 × 10^0^ and 2 × 10^1^ CFU/ml) inoculated in 10 g of PIF. The assay was able to detect as few as 2 cells of *C. sakazakii*/10 g of PIF sample after 6 h of pre-enrichment incubation with an assay time of 2 h 30 min. The assay was assessed for cross-reactivity with other bacterial strains and exhibited strong specificity to *C. sakazakii*. Moreover, the assay method was applied to the detection of *C. sakazakii* in PIF without pre-enrichment steps, and the results were compared with INC-ELISA and RT-PCR. The developed method was able to detect *C. sakazakii* in spiked PIF without pre-enrichment, whereas INC-ELISA failed to detect *C. sakazakii*. In addition, when compared with the results obtained with RT-PCR, our developed assay required lesser detection time. The developed assay was also not susceptible to any effect of the food matrix or background contaminant microflora.

Globalization of the food supply has expanded the range of foodborne pathogens. In recent years, special concerns have been raised about the safety of infant milk powder-based food products contaminated by *Cronobacter sakazakii (C. sakazakii*) following several incidents of foodborne illness associated with produce. *C. sakazakii* has been found in a variety of dry foods, including powdered infant formula (PIF), skim milk powder, herbal teas, and starches^1^. Although *Cronobacter* illnesses are rare, they are frequently lethal in infants and can be serious among immunocompromised individuals and the elderly^2^. Indeed, this microbe, which was first recognized as “yellow-pigmented *Enterobacter cloacae*” in 1961, poses a serious health risk to infants^3^. Surveys and reports have confirmed lower frequencies of reported cases of infant infection by *C. sakazakii* than other foodborne pathogens. In South Korea, no case of infant infection associated with *C. sakazakii* has been reported thus far, except for two local hospital notices published without documentation or clinical confirmation. However, antibiotic susceptibility tests have revealed that *C. sakazakii* shows occasional antibiotic resistance^4^. Because there are limited antibiotic therapies against this hazardous pathogen, there is an urgent need for novel alternative biocontrol or detection methods for *C. sakazakii*.

Potential biological threats to our health and economy have established the importance of foodborne pathogen detection methods^5^. In recent decades, various methodologies have been developed to detect these pathogens, especially *C. sakazakii*, in contaminated food and feed. However, the low infective dose required for *C. sakazakii* infection makes control of this pathogen difficult, and thus strict monitoring is required^6^. Although only a few species of *Cronobacter*, such as *C. sakazakii, Cronobacter malonaticus (C. malonaticus)*, and *Cronobacter turicensis (C. turicensis*), have been linked with human illnesses, current international microbiological standards require complete eradication of *Cronobacter* species from PIF, further demonstrating the need for the development of specific, sensitive, and rapid identification methods to secure adequate food safety^1^.

Classical techniques used for the detection of bacterial contamination are based on microbiological techniques such as swabbing, pre-enrichment, and selective plating, which are reliable approaches for pathogen detection and are standard methods used by regulatory agencies^7^. However, analysis of bacterial contamination using these classical methods is time consuming and labor intensive. In addition, real-time polymerase chain reaction (RT-PCR) and/or PCR-based methods can detect bacterial pathogens within a relatively short time^8^,^9^. However, issues such as primer design, PCR-mediated inhibition of complex food matrices, and high equipment costs are barriers to PCR detection becoming a routine procedure for pathogen identification^9^. There are some commercial detection kits available for the detection of *Cronobacter* species, but most are PCR-based methods (Table 1). Therefore, further development of cost-effective, simple, and rapid detection methods for the routine detection of *C. sakazakii* remains important.

An alternative and effective approach to overcome complex PCR-based methods involves immunoassays such as enzyme-linked immunosorbent assays (ELISAs) based on liposomes^10^,^11^. Previously, we reported sandwich ELISA and indirect non-competitive ELISA (INC-ELISA) for the detection of *Cronobacter muytjensii (C. muytjensii*) in pure culture^12^,^13^. Liposomes are highly versatile structures for research and therapeutics as well as analytical and immunological applications. Liposome-based immunoassays have applicability in various areas of food chemistry, food microbiology, nano-biotechnology, and diagnostic or clinical microbiology, such as for the detection of foodborne pathogens, toxins, and hazardous components present in food as well as the human body^14^.

Recently, we developed an immunoliposome and immunomagnetic nanoparticle-based immunoassay for the detection of *C. sakazakii* in bacterial cultures^15^. Immunomagnetic separation, which uses solid phase-bound constituents such as antibody-conjugated magnetic nanoparticles (immunomagnetic nanoparticles), is suitable for the isolation of target bacteria from fluids^16^. Immunomagnetic (nano) particle-bound target bacteria are separated from the mixed suspension by a strong magnetic force and then concentrated from a large volume of crude culture solution into a smaller volume of purified culture solution. This principle suggests that the combination of fluorescence-encapsulated, antibody-tagged liposomes (immunoliposomes) and immunomagnetic nanoparticles may enhance the detection capability more than 10-fold as compared with that of other liposome-based assays previously developed for pathogen detection^15^,^16^.

Hence, as a part of our continuing efforts to confirm the practical application of the developed assay for rapid and sensitive detection of foodborne pathogens, we optimized and validated an efficient, rapid, and sensitive immunoliposome and immunomagnetic nanoparticle-based immunoassay method and confirmed its practical applicability for the detection of *C. sakazakii* in artificially contaminated PIF samples by comparing its efficacy with other conventional detection methods such as ELISA and RT-PCR. The main aims of the current study were to determine the applicability of the developed assay in food samples, demonstrate its reduced detection time (food sample enrichment period), and assess its possible use in detection without the requirement for an enrichment step.

## Materials and Methods

### Chemicals and reagents

1,2-Dipalmitoyl-*sn*-glycero-3-[phospho-rac-(1-glycerol)] (DPPG), 1,2-dipalmitoyl-*sn*-glycero-3-phosphoethanolamine (DPPE), and 1,2-dipalmitoyl-*sn*-glycero-3-phosphocholine (DPPC) were purchased from Avanti Polar Lipids (Alabaster, AL, USA). Sulforhodamine B (SRB) was purchased from Molecular Probes (Eugene, OR, USA). Nutrient agar (NA), nutrient broth (NB), skim milk, and bacto peptone were purchased from Difco Laboratories Inc. (Detroit, MI, USA). Buffered peptone water (BPW) was purchased from Oxoid Ltd. (Basingstoke, Hampshire, England). Violet red bile glucose (VRBG) agar was purchased from MB Cell (Los Angeles, CA, USA). *n*-Octyl-*β*-D-glucopyranoside (OG), cholesterol, sodium azide, sodium chloride, potassium phosphate monobasic, potassium phosphate dibasic, Tris, alkaline phosphatase yellow liquid substrate *p-*nitrophenyl phosphate (*p*NPP), Tween 20, sodium hydroxide, and rabbit gamma globulin were purchased from Sigma-Aldrich (St. Louis, MO, USA). Carboxyl magnetic iron oxide (Fe_3_O_4_) particles, 1-ethyl-3-(3-dimethylaminopropyl) carbodiimide (EDAC)/N-hydroxysuccinimide (NHS), and reaction buffers for conjugation were purchased from Ocean Nanotech (Springdale, AR, USA). The magnetic particle separator was purchased from Dynal Inc. (Lake Success, NY, USA). Phosphatase-labeled goat anti-rabbit immunoglobulin G (IgG) was purchased from Kierkegaard & Perry Laboratories, Inc. (Gaithersburg, MD, USA). *n*-Succinimidyl-*s*-acetylthioacetate (SATA) was obtained from Pierce (Rockford, MD, USA). Ninety six-well microtiter plates were purchased from SPL Life Sciences (Pocheon, Korea). Primers (Crono-F and Crono-R) and probe (Crono-P) used for RT-PCR were purchased from Microgen (Daejon, Korea). QiaAmp DNA extraction mini kit was purchased from Qiagen (Hilden, Germany). Taq polymerase was obtained from Invitrogen (Carlsbad, CA, USA). IQ supermix was purchased from Bio-rad (Hercules, CA, USA).

### Bacteria and culture conditions

The bacterial strain used in this study to produce antibody was *C. sakazakii* (ATCC 29544), which was obtained from the American Type Culture Collection (ATCC, Manassas, VA, USA). *Bacillus cereus (B. cereus*) (Korean Culture Center of Microorganisms: KCCM 40935), *Citrobacter freundii (C. freundii*) (ATCC 8090), *Enterobacter aerogenes (E. aerogenes*) (ATCC 15038), and *Salmonella* Enteritidis (*S.* Enteritidis) (ATCC 4931) were used to check cross-reactivity and microbial background effect of the developed method. All strains used in this study were cultured in NB for 18–20 h at 37 °C on a shaking incubator (150 rpm).

### Antibody production

Polyclonal antibody against *C. sakazakii*, termed anti-*C. sakazakii* IgG, was produced according to our previously developed procedure^12^, which was used in this study for validation of the detection method. Animal use protocol was reviewed by the committee members of Yeungnam University and approved by Korea Food and Drug Administration, Republic of Korea (Animal Ethics License No. 2013-012 and 2012-010). All methods were performed in accordance with the relevant guidelines and regulations.

### Preparation of SRB-encapsulated, anti-*C. sakazakii* IgG-tagged immunoliposomes

Fluorescent dye-encapsulated liposomes were prepared via a reversed-phase evaporation method with minor modifications using cholesterol (40.9 μmol), DPPG (4.2 μmol), DPPE (7.2 μmol), DPPC (40.3 μmol), SATA (4.3 μmol), and SRB (100 mM), followed by conjugation with anti*-C. sakazakii* IgG as previously described^15^,^17^.

### Determination of particle size, polydispersity index (PDI), and zeta potential of SRB-encapsulated liposomes

The average diameter, PDI, and zeta potential of liposomes were measured by dynamic light scattering (DLS) with a Zetasizer Nano ZS particle analyzer (Malvern Instruments Ltd., Worcestershire, UK) at room temperature. The liposomal suspension was adequately diluted with 0.02 M Tris-buffered saline (TBS pH 7.0) prior to measurement to adjust the intensity. The PDI was also determined as a measurement of the level of homogeneity of particle size.

### Preparation of immune-functionalized magnetic nanoparticles

**Figure 1:**
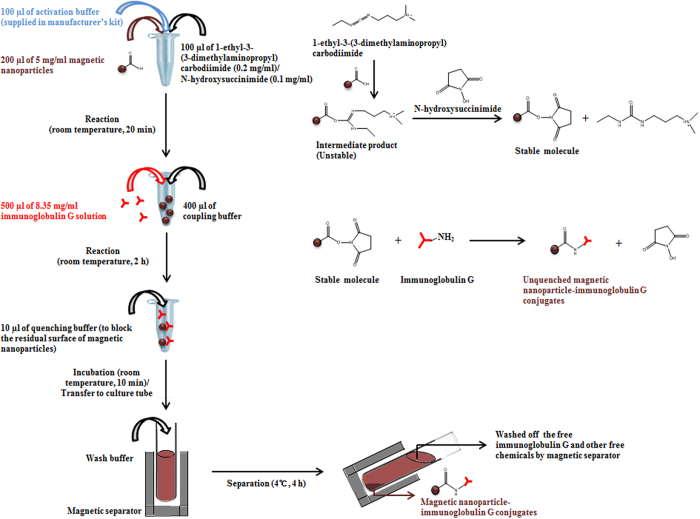
Schematic representation of the immunoglobulin G and magnetic nanoparticles conjugation reaction.

Conjugation of iron oxide magnetic nanoparticles (200 μl of 5 mg/ml) with anti-*C. sakazakii* IgG (500 μl of 8.35 mg/ml) was performed as per the manufacturer’s instructions and also according to our previously optimized procedure^15^. A detailed outline of the procedure and chemical reaction is shown in Fig. 1. After conjugation of the magnetic nanoparticles with anti-*C. sakazakii* IgG, the conjugation efficiency of the immunomagnetic complex was determined by calculating the difference between the initial amount of anti-*C. sakazakii* IgG and the amount of free anti-*C. sakazakii* IgG using the standard calibration curve of rabbit gamma globulin. The prepared immunomagnetic nanoparticles (magnetic nanoparticles conjugated with anti-*C. sakazakii* IgG) were stored in a refrigerator until further use.

### Artificial inoculation of *C. sakazakii* into PIF samples

**Figure 2:**
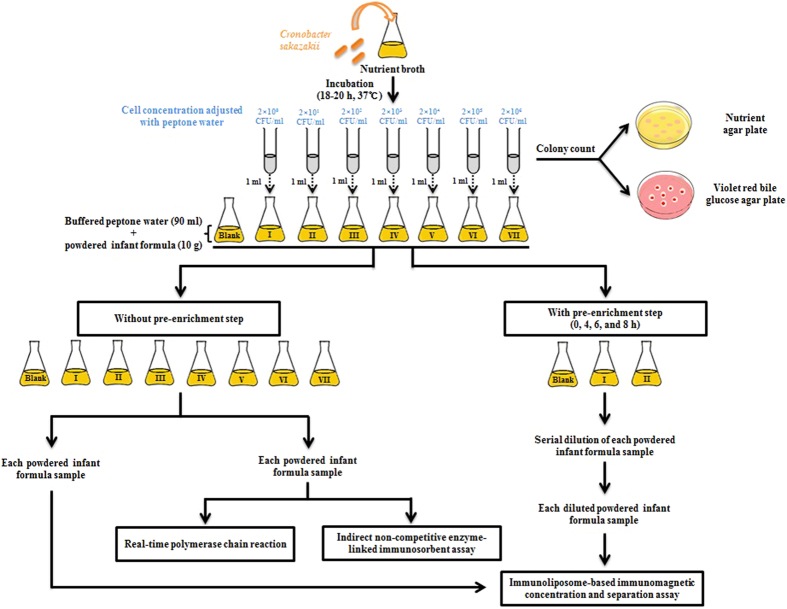
Schematic representation of the overall procedure for the detection of *Cronobacter sakazakii*.

The stock bacterial suspension of *C. sakazakii* was cultured in NB at 37 °C for 18–20 h, after which the culture was serially diluted with 0.1% peptone water (w/v) to prepare the desired concentrations of *C. sakazakii*, which were used in further experiments. For each parameter, 10 g of PIF sample was aseptically added to a flask, mixed with 90 ml of 2% BPW (w/v), and separately spiked with 1 ml culture of *C. sakazakii* at various concentrations, followed by mixing. Then, these contaminated PIF samples were tested using the developed immunoliposome-based immunomagnetic concentration and separation assay, INC-ELISA, and RT-PCR for validation of *C. sakazakii* detection. In another set of experiments, 10 g of PIF sample was similarly contaminated with 1 ml of culture of *C. sakazakii* at concentrations of 2 × 10^0^ and 2 × 10^1^ CFU/ml, followed by pre-enrichment incubation at 37 °C for 0, 4, 6, and 8 h, after which these PIF samples were tested using the immunoliposome-based immunomagnetic concentration and separation assay. A detailed experimental scheme of the overall process is presented in Fig. 2. Simultaneously, to confirm the results of the developed immunoliposome-based immunomagnetic concentration and separation assay for *C. sakazakii* detection, a conventional standard plate count using the spreading method on NA and VRBG agar plates was performed, and the detection limit of the developed assay was reconfirmed. Commercially available PIF without *C. sakazakii* cell inoculum was used as a blank control. All experiments were performed in triplicate to verify the reliability and reproducibility of the results.

### Detection of *C. sakazakii* in artificially contaminated PIF samples using the immunoliposome-based immunomagnetic concentration and separation assay

**Figure 3:**
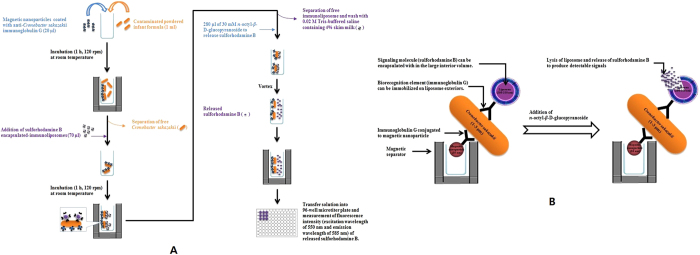
Procedure of the immunoliposome-based immunomagnetic concentration and separation assay (**A**) and schematic diagram for the production of fluorescent signals in the immunoliposome-based immunomagnetic concentration and separation assay for the detection of *Cronobacter sakazakii* (**B**).

The assay was performed by separately dispensing 1 ml of pre-enriched and non-pre-enriched samples of artificially contaminated PIF into separate glass tubes, followed by the addition of 20 μl of immunomagnetic nanoparticles and incubation at room temperature for 1 h with gentle shaking. Then, the mixture was placed in a magnetic separator for 3 min, after which the supernatant was removed. *C. sakazakii*-bound immunomagnetic nanoparticle complexes were washed twice with 1 ml of 0.01 M phosphate-buffered saline (PBS), containing 0.15 M NaCl, 0.01% NaN_3_, and 0.05% Tween 20, and once with 1 ml of 0.02 M TBS, containing 4% skim milk, using a magnetic separator for 3 min. Then, 70 μl of 20-fold diluted anti-*C. sakazakii* IgG-tagged liposome (immunoliposomes) solution was added, followed by incubation for 1 h at room temperature with gentle shaking to produce the immunomagnetic nanoparticles-*C. sakazakii*-immunoliposomes complex. To remove unbound immunoliposomes and reduce non-specific binding, the complex was washed with solution consisting of 0.02 M TBS and 4% skim milk. Then, 30 mM OG solution (280 μl) was added to the immunomagnetic nanoparticles-*C. sakazakii*-immunoliposomes complexes, followed by vortexing, after which the supernatant (260 μl) containing SRB was transferred to a 96-well microtiter plate. Finally, the fluorescence intensity was measured at an excitation wavelength of 550 nm and an emission wavelength of 585 nm using a microplate reader (Infinite M200, Tecan, Mannedorf, Switzerland). A schematic of the principle/format of the developed assay is presented in Fig. 3A,B.

The assay procedure consisted of three important steps: (I) immunomagnetic concentration and separation of the contaminant *C. sakazakii* present in PIF, (II) reaction of immunoliposome particles with the separated *C. sakazakii* attached to immunomagnetic nanoparticles, and (III) fluorescent signal generation from SRB released after lysis of the immunoliposome particles (Fig. 3A). In the first step of assay development, the contaminated PIF sample containing *C. sakazakii* was concentrated and separated using immunomagnetic nanoparticles. After this step, the concentrated *C. sakazakii* bound to the immunomagnetic nanoparticles was added to immunoliposome particles, resulting in a heavier complex composed of immunomagnetic nanoparticles-*C. sakazakii*-immunoliposomes. The complexes were again passed through a magnetic field to remove free immunoliposome particles. To the complexes, OG was added as a detergent to lyse the immunoliposome particles to generate detectable signals from the complexes. The addition of 30 mM OG to the complexes resulted in lysis of the immunoliposomes and produced fluorescent signals, which were measured at an excitation wavelength of 550 nm and an emission wavelength of 585 nm (Fig. 3A). A comprehensive schematic of the mechanism of the assay is presented in Fig. 3B. The signal intensities of both the test and blank samples were measured and calculated for positive/negative (P/N) values.

### Comparison of the developed method with INC-ELISA in a PIF samples

The immunoliposome-based immunomagnetic concentration and separation assay developed in this study was compared with an INC-ELISA previously developed for the detection of *C. sakazakii* to visualize the detection sensitivity and capability of the developed assay without pre-enrichment. The INC-ELISA method was adopted for this assay with slight modifications^13^. Briefly, PIF samples without pre-enrichment and artificially contaminated with different concentrations of *C. sakazakii* in the range of 10^0^–10^6^ CFU/ml were used to evaluate the detection capability of the developed assay. Samples were then coated onto a 96-well microtiter plate, followed by incubation at 4 °C overnight, washing three times with 0.01 M PBS (pH 7.0), and blocking with 200 μl of 5% skim milk for 2 h at 37 °C. The plate was then washed with 0.01 M PBS containing 0.05% Tween 20 (PBST). Then, anti-*C. sakazakii* IgG was added to each well, followed by incubation at 37 °C for 1 h. The plate was washed again with 0.01 M PBST, after which phosphatase-labeled goat anti-rabbit IgG was added, followed by incubation at 37 °C for 1 h. After re-washing with PBST, 50 μl of *p*NPP liquid substrate was added to each well for a 30 min enzyme-substrate reaction. Thereafter, 50 μl of 1 M NaOH was added to each well to stop the reaction, after which the yellow color produced in positive reaction wells was measured at an absorbance wavelength of 405 nm using a microplate reader. Each experiment was performed in triplicate.

### Comparison of the developed method with RT-PCR

The detection sensitivity for *C. sakazakii* using RT-PCR was compared with our developed immunoliposome-based immunomagnetic concentration and separation assay in pure culture and artificially contaminated PIF samples. Genomic DNA was extracted and purified from an overnight culture of different concentrations (10^0^–10^8^ CFU/ml) of *C. sakazakii*. A volume of 1 ml of serially diluted bacterial culture (in 0.85% NaCl) was used for extracting DNA using the QiaAmp DNA mini kit. All steps were followed according to the manufacturer’s instructions using a protocol for bacteria. Bacterial cultures of 1 × 10^8^ CFU/ml showed a DNA concentration of approximately 27.0 ng/ml. PCR was run in duplicate under optimum cycling conditions as described below. The RT-PCR method of Chen *et al.*^18^ was adopted for the detection of *C. sakazakii* in artificially contaminated PIF samples. Each experiment was performed in triplicate.

### Primers and PCR conditions

Based on the sequence data generated and the results of the online BLAST analysis, a pair of primers, Crono-F (5′-GGGATATTGTCCCCTGAAACAG-3′), Crono-R (5′-CGAGAATAAGCCGCGCATT-3′), and Crono-P (5′-6FAM-AGAGTAGTAGTTGTAGAGGCCGTGCTTCCGAAAG-TAMRA-3′) were designed to amplify a 78 bp *C. sakazakii*-specific fragment. A PCR mixture (25 μl) contained 12.5 μl IQ supermix (100 mM KCl, 40 mM Tris-HCl (pH 8.4), 0.4 mM each dNTP, 50 U/ml iTaq DNA polymerase, and 6 mM MgCl_2_), 900 nM of each Crono-F and Crono-R primers, 250 nM of Crono-P probe, 0.5 U of Taq polymerase, and 2 μl of sample DNA. PCR was performed in an a 48-well thermal cycler (Stepone real-time PCR system, Applied Biosystems, Singapore) using the following conditions: one cycle of denaturation at 95 °C for 3 min, followed by 40 cycles at 95 °C for 15 s, 52 °C for 20 s, and 72 °C for 30 s. The fluorescence was recorded at the end of each annealing step.

### Cross-reactivity performance assay

To test whether the developed assay system was able to detect *C. sakazakii* specifically in real food samples, spiked PIF samples were made by inoculating other genera such as *C. freundii* and *E. aerogenes*, and the cross-reactivity of the developed method was examined against *C. sakazakii* as a target pathogen. The analytical procedure was similar to that described above. Each strain was individually cultured at 37 °C for 18–20 h in NB media with shaking (150 rpm). After 18–20 h, pure cultures of each strain were adjusted to 10^8^ CFU/ml with 0.1% peptone water. The assay procedure was similar to that described under the section titled “Detection of *C. sakazakii* in artificially contaminated PIF samples using the immunoliposome-based immunomagnetic concentration and separation assay” in the Materials and Methods section. For the blank, market PIF without any inoculation was used.

### Effect of background microflora

Specific criteria are applied for the analysis of food and water samples; one key aspect is that the target microorganisms must be detected amidst background microflora. To evaluate the effect of background microflora, PIF samples were collectively spiked with other bacterial strains belonging to different genera, including *B. cereus*, *C. freundii*, and *S.* Enteritidis, at a concentration of 10^8^ CFU/ml with different concentrations of *C. sakazakii* (2 × 10^3^, 2 × 10^5^, and 2 × 10^8^ CFU/ml), analyzed using the developed immunoliposome-based immunomagnetic concentration and separation assay.

### Statistical analysis

The detection limit was calculated as the average value of absorbance at zero concentration (blank) with three standard deviations^19^. Statistical analysis was performed using the IBM SPSS 19 program (SPSS Inc., Chicago, IL., USA). A multiple Duncan’s test was applied and *P* value < 0.05 was considered significant for each of the statistical parameters.

## Results and Discussion

### Characterization of liposomes

In the present study, we successfully prepared SRB dye-encapsulated liposome particles using the reversed-phase evaporation method. An important aspect of the preparation of liposomes, from both fundamental and technological viewpoints, is the production of a desired particle size with a narrow size distribution and good stability^20^. The mean particle size, zeta potential, and PDI of prepared liposomes are presented in Table 2.

Previously, our research group confirmed that liposome particles could remain stable for up to 100 days^21^. The size and PDI of SRB-encapsulated liposome particles were analyzed using DLS and were found to be 244.3 nm and 0.246, respectively (Table 2). A narrow size range for SRB-encapsulated liposomes is extremely important to obtain good sensitivity and a suitable detection range for the test procedure^22^. Extrusion of the liposome preparations through polycarbonate filters reduced the size heterogeneity, and liposomes that were between 230 and 250 nm were used in all subsequent experiments. Prepared liposomes with lower PDI (less than 1) values are considered monodisperse liposome particles with good stability^23^. A high PDI indicates nanoparticles with a low homogeneity in size distribution^24^. Liposome particles of a similar size and PDI range were also shown to be stable^24^. Our results showed that the prepared liposomes remained stable for long periods of time, which indicates that our newly developed liposomal formulation meets the requirements for an effective detection system.

The magnitude of the zeta potential indicates the potential stability of the colloidal system. If all of the particles in suspension have a large negative or positive zeta potential, then they will tend to repel each other, and there will be little tendency for the particles to come together. In contrast, if the particles have low zeta potential values, there will be no force preventing them from coming together and flocculating. In the present study, the zeta potential observed in several lots of our SRB-encapsulated liposome particles, which was found to be −42 ± 7.72 mV (Table 2), indicated a negative surface charge. These values are in good agreement with several previous studies^24^,^25^,^26^. This negative surface charge was imparted primarily by the phosphatidic acid necessary for antibody binding to the liposome particles^25^ that was present in the phospholipid products and the molar concentration used in preparation. Valuable zeta potential information about a liposome preparation can help to predict the fate of the liposomes *in vivo*, and modification of the liposome surface can also be monitored by measurement of the zeta potential.

SRB-encapsulated liposomes were used as signal-generating agents for the detection of *C. sakazakii*. Release of liposome contents as a biosensor of loss of liposome integrity has been reported previously. Materials encapsulated by liposomes in these cases were either fluorescent molecules or redox couples^27^,^28^. In the present study, SRB-encapsulated liposomes represented a similar approach to better accommodate the requirements for integrity of the prepared liposome particles in a portable sensing platform. In the present work, the fluorescence intensity of the SRB-encapsulated liposomes was measured before and after liposome rupture. Addition of 930 mM OG as a detergent to rupture the liposome particles caused the encapsulated SRB dye to leak out of the liposome particles, resulting in higher fluorescent signals compared to those obtained before liposome rupture (Fig. S1). These results confirmed the structural integrity of the SRB-encapsulated liposomes.

### Conjugation of anti-*C. sakazakii* IgG and magnetic nanoparticles

In this study, magnetic nanoparticles of 30 nm in diameter with a zeta potential of −53 ± 9.89 mV (Table 2) were conjugated to antibody made in our laboratory against *C. sakazakii* to prepare immunomagnetic nanoparticles. In general, magnetic nanoparticles are widely used for detection purposes due to their easy separation and/or enrichment from complicated food matrices simply by applying a magnetic field. Therefore, functionalized magnetic nanoparticles are applicable to the specific and/or non-specific detection of notorious bacterial pathogens, toxins, and related products^29^,^30^. A schematic diagram and chemical reaction illustrating the process of conjugation of anti-*C. sakazakii* IgG to the magnetic nanoparticles is presented in Fig. 1, where carboxyl (COOH) functionalized magnetic nanoparticles are activated with EDAC and NHS. An amine-reactive NHS ester on the magnetic nanoparticle reacts with primary amines on the antibody to yield IgG-conjugated magnetic nanoparticles.

**Figure 4:**
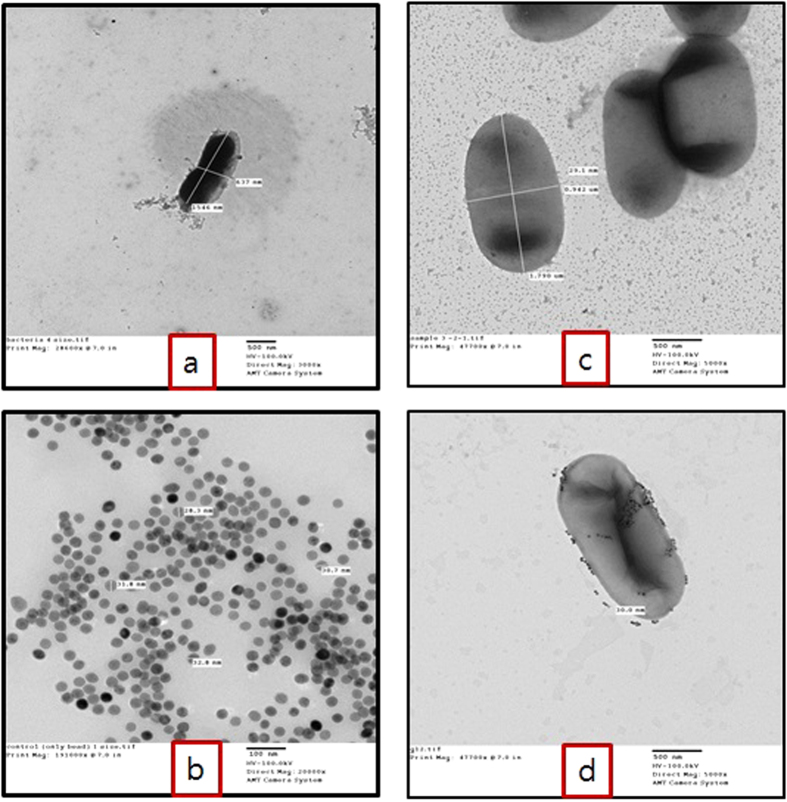
Transmission electron microscope image (**a**) of a single cell of *Cronobacter sakazakii*; (**b**) of magnetic nanoparticles; and (**c,d**) of *Cronobacter sakazakii* cells bound to multiple magnetic nanoparticles.

The hydrodynamic size of the magnetic nanoparticles and the IgG-conjugated magnetic nanoparticles dispersed in distilled water was measured using a Zetasizer Nano ZS particle analyzer. The size of the magnetic nanoparticles obtained using transmission electron microscopy (TEM) was close to 30 nm (Fig. 4b), whereas the hydrodynamic size (diameter) and zeta potential were found to be 163.9 nm and −53 ± 9.89 mV, respectively (Table 2). The hydrodynamic size was larger due to the thickness of the hydrated layer on the surface of magnetic nanoparticles in distilled water. Moreover, the average hydrodynamic size was again increased to 373.4 nm after antibody conjugation on the surface of the magnetic nanoparticles (Table 2), likely due to the average hydrodynamic size of anti-*C. sakazakii* IgG. Similar findings were also observed by Chen *et al.*^31^ during the quantification of a tumor marker carbohydrate antigen using superparamagnetic nanoparticles. Magnetic nanoparticles exhibited a PDI of 0.289 (Table 2), indicating their strong stability and the best particle size distribution of all evaluated systems. Moreover, we measured the zeta potential of nanoparticles in distilled water (pH 7.4) to test the difference in the surface charge of magnetic nanoparticles before and after antibody conjugation. As shown in Table 2, the particles had a negative surface charge prior to antibody coating and maintained a negative surface charge after antibody conjugation, with zeta potentials of −53 ± 9.89 mV and −46 ± 4.37 mV, respectively. The negative surface charge of the free magnetic nanoparticles was due to the carboxyl groups that were necessary for binding the antibody. The decrease in surface potential was due to antibody conjugation and was also consistent with an increase in size. Similar trends were also reported by Chen *et al.*^31^.

A commonly used method for conjugating antibodies to magnetic particles is covalent coupling^32^. Although this method is simple and gentle, the adsorbed antibody is easily affected by minor changes in pH, monodispersity, and cross-linking time. Hydrogen bonds and van der Waals forces also contribute to the stability of the system^33^,^34^. The conjugation efficiency of the magnetic nanoparticles with antibody was determined by generating a standard curve for rabbit gamma globulin (as a standard protein) by measuring its absorbance at various concentrations. The amount of antibody conjugated to the magnetic nanoparticles was calculated based on the difference between the initial amount of anti-*C. sakazakii* IgG and the amount of free anti-*C. sakazakii* IgG^35^. In the initial stage of conjugation, 8.35 ± 0.00 mg/ml of anti-*C. sakazakii* IgG was used, after which the conjugation mixture underwent several chemical reactions. In the final stage of the conjugation reaction, the amount of unbound antibody was analyzed with the Bradford assay^35^. The concentration of unbound antibody in the supernatant (free antibody after the magnetic separation step) was determined to be 0.96 ± 0.01 mg/ml, confirming that immunomagnetic nanoparticles had a bound antibody concentration of 7.39 ± 0.59 mg/ml (Table 3). These results confirmed that the conjugation efficiency (%) of the antibody to magnetic nanoparticles was 88.5% (Table 3). All experiments were performed in triplicate, and the coefficient of variation was less than 15%. Similarly, Tu *et al.*^35^ reported a 76–96% bound antibody concentration when smaller magnetic particles were used, compared with larger magnetic particles. The microspheres showed a higher efficiency for the capture of microorganisms and other objects due to their large surface area. Additionally, microspheres showed good stability in solution due to their small particle size^36^,^37^. Thus, small immunomagnetic nanoparticles exhibit good magnetic separation efficiency.

The capture efficiency of immunomagnetic nanoparticles with *C. sakazakii* bacterial cells was determined by morphological examination using TEM. This study verified the attachment of target bacterial cells (*C. sakazakii*) to immunomagnetic nanoparticles, confirming successful development of the immunomagnetic nanoparticles. The TEM images clearly showed that multiple immunomagnetic nanoparticles bound to the surface of a single bacterial cell and enhanced the level of applied magnetic force for better attachment and detection capabilities (Fig. 4). For the biological binding reaction, small particles with a high surface/volume ratio and high mobility have many opportunities to interact with bacterial cells in solution, resulting in a high capture efficiency as previously reported^38^. Similarly, Shan *et al.*^33^ observed images of immunomagnetic beads with a high capture efficiency of 94.21% for the target pathogen *Listeria monocytogenes (L. monocytogenes*).

Rapid magnetic separation of bacteria with an immunomagnetic separation assay must overcome a substantial amount of resistance force as it moves through the separation solution toward the magnet^39^. The capture efficiency can be affected by the magnetic intensity as well as by the various separation media, including PBS buffer. In our work, we used immunomagnetic nanoparticles with PBS as the separation and storing buffer. Shan *et al.*^33^ showed that capture efficiency was the highest when PBS buffer was selected as the separation medium because there is no matrix interference in PBS buffer. The assay method developed in this study was optimized based on these findings.

### Detection of *C. sakazakii* in artificially spiked PIF samples

**Figure 5:**
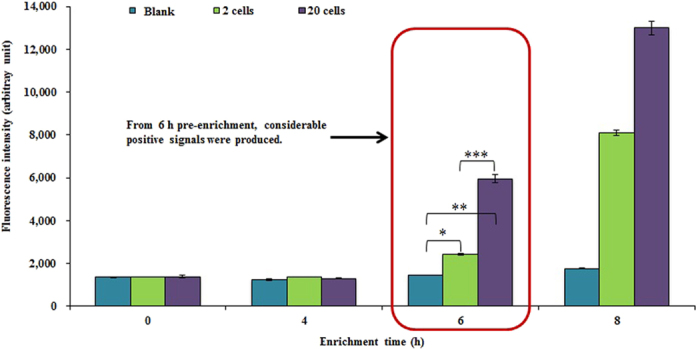
Detection of *Cronobacter sakazakii* in powdered infant formula by the immunoliposome-based immunomagnetic concentration and separation assay according to increasing enrichment time. All experiments were conducted three times, and the data represent the mean ± standard deviation. *Significantly different (*P* < 0.05) compared with blank at 6 h pre-enrichment; **significantly different (*P* < 0.05) compared with blank at 6 h pre-enrichment; ***significantly different (*P* < 0.05) compared with 2 cells after 6 h pre-enrichment.

In this study, the developed immunoliposome-based immunomagnetic concentration and separation assay was used to check for the presence of *C. sakazakii* in PIF purchased from a grocery store (as a control) and other PIF samples artificially contaminated with specific levels of *C. sakazakii*. As shown in Fig. 5, the detection limit of the developed assay against *C. sakazakii* was 2 cells/10 g of PIF after 6 h of pre-enrichment incubation. In addition, when the tests were performed using artificially contaminated PIF samples with 8 h of pre-enrichment incubation, the developed assay exhibited a higher positive fluorescent signal for the PIF sample containing *C. sakazakii* at a concentration of 2 cells/10 g of PIF (Fig. 5). The test was also performed after 4 h of pre-enrichment incubation, and the results showed that detection of 2 cells/10 g was not statistically significant (Fig. 5). Eventually, to reduce assay time, contaminated PIF samples with 6 h of pre-enrichment incubation were tested, and the developed method could significantly detect an initial number of *C. sakazakii* as low as 2 cells/10 g of PIF sample. Based on these time-reducing experiments, the detection limit of the developed assay against *C. sakazakii* in artificially contaminated PIF samples was determined to be 2 cells/10 g of PIF after 6 h of pre-enrichment incubation.

To confirm the results of the developed immunoliposome-based immunomagnetic concentration and separation assay for *C. sakazakii* detection, a conventional standard plate count technique based on spreading on NA and VRBG agar plates was used, and the detection limit was reconfirmed. The artificially contaminated PIF samples (with 2 cells/10 g of PIF) after 6 h of pre-enrichment incubation showed *C. sakazakii* counts near 10^3–4^ CFU/ml. Similarly, Blazkova *et al.*^40^ developed a method using an immunochromatographic strip for the detection of *Cronobacter* species, and the method was able to detect less than 10 cells/10 g of PIF. In the present study, all PIF samples artificially contaminated with *C. sakazakii* were positively detected after 6 h of pre-enrichment and 2 h 30 min of assay time. Importantly, detection and confirmation of this pathogen with the official culture-based method takes up to 3–5 days^41^, thus confirming the improved applicability of the developed assay for the simple and sensitive detection of *C. sakazakii* in food samples in a shorter amount of time.

Recently, Yu *et al.*^42^ developed a new detection method for *Staphylococcus aureus (S. aureus*) in milk samples by coupling immunomagnetic separation with the enzyme-linked cell wall-binding domain of lysin, which can bind to *S. aureus* with high affinity. The overall method takes only 1.5 h and shows a similar detection limit (10^3^ CFU/ml) in spiked milk samples as observed in our study using the developed immunoliposome-based immunomagnetic concentration and separation assay. Although the assay method reported by Yu *et al.*^42^ demonstrated sensitive detection of *S. aureus* in spiked milk samples with a shorter testing time, our developed method showed higher detection stability through the use of liposome particles as sensor molecules instead of a horseradish peroxidase enzyme label, confirming that our newly developed method has better industrial applicability for pathogen detection. In our earlier report, we confirmed that liposomes prepared with this method could remain stable for up to 100 days^21^.

### Comparison of the developed method with INC-ELISA

**Figure 6:**
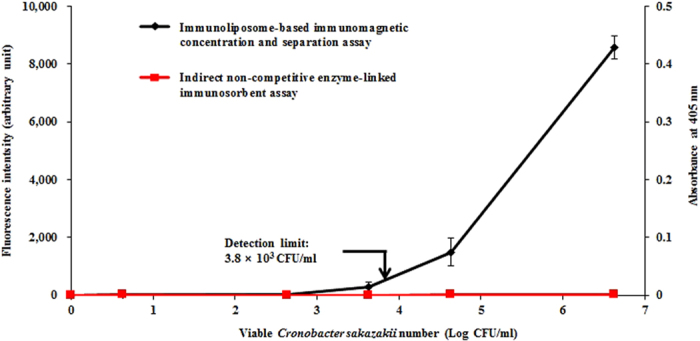
Comparison of the immunoliposome-based immunomagnetic concentration and separation assay with an indirect non-competitive enzyme-linked immunosorbent assay in a powdered infant formula without pre-enrichment step. All experiments were conducted three times, and data represent the mean ± standard deviation. The coefficient of variation for fluorescence intensity (n = 6) was below 15%.

To confirm detection quality, pre-enrichment incubation, and similarity, the immunoliposome-based immunomagnetic concentration and separation assay was compared with our previously developed INC-ELISA. In the comparison of the immunoliposome-based immunomagnetic concentration and separation assay with the INC-ELISA, the developed assay was tested using PIF samples artificially contaminated with various concentrations of *C. sakazakii* (in the range of 10^0^–10^6^ CFU/ml) without a pre-enrichment step. As a result, the developed assay was able to detect *C. sakazakii*, without pre-enrichment at a concentration of approximately 10^3^ CFU/ml in 10% PIF solution (w/v) (Fig. 6). However, the INC-ELISA method was unable to detect *C. sakazakii* cells in contaminated PIF samples without the pre-enrichment step, even at the highest cell concentration (Fig. 6), possibly due to its bacterial cell concentration step, thus confirming the effectiveness of our immunoliposome-based immunomagnetic concentration and separation assay for pathogen detection. These findings reinforce the notion that the developed immunoliposome-based immunomagnetic concentration and separation assay provides improved detection ability within a shorter assay performance time (2 h 30 min) as well as a favorable cost by omitting pre-enrichment steps needed for INC-ELISA. In addition, ELISA methods require certain enrichment procedures that take a minimum of 8–10 h, which is considered a long and labor-intensive analysis^43^.

The detection limit of the immunoliposome-based immunomagnetic concentration and separation assay in the selected food matrix (PIF) was found to be similar (3.8 × 10^3^ CFU/ml) to that observed in the pure *C. sakazakii* culture^15^, indicating that the developed method is not affected by competing molecules in the food system and has significant potential for detecting *C. sakazakii* in real PIF samples. Similar findings were also observed by Cho and Irudayaraj^44^ for the detection of *L. monocytogenes*. However, in that case, enzyme-based immunomagnetic detection was used, whereas in the current study, we developed a method based on immunoliposome particles. To demonstrate the reproducibility and acceptable precision of an assay, the coefficient of variation should be less than 15–20%^45^. In all of our studies, the coefficient of variation was found to be under this limit.

### Comparison of the developed method with RT-PCR

**Figure 7:**
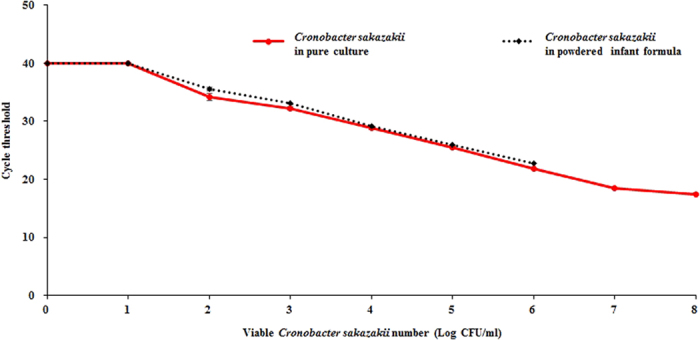
Detection of *Cronobacter sakazakii* by real-time polymerase chain reaction in pure culture and powdered infant formula. All experiments were conducted three times, and data represent the mean ± standard deviation.

The detection sensitivity and applicability of RT-PCR for detecting *C. sakazakii* in contaminated PIF samples (without pre-enrichment) was checked and compared with our developed immunoliposome-based immunomagnetic concentration and separation assay. In pure culture, RT-PCR showed a detection limit of 10^2^ CFU/ml (Fig. 7), which was also found to be similar in contaminated PIF samples without pre-enrichment (Fig. 7). Our developed immunoliposome-based immunomagnetic concentration and separation assay required less detection time (2 h 30 min) for *C. sakazakii* in contaminated PIF samples, while the RT-PCR assay employs a DNA extraction step, which requires 3–4 h of additional PCR run time with a minimum of 3 h. Therefore, we confirm that our developed immunoliposome-based immunomagnetic concentration and separation assay exhibits a shorter time requirement. There are several advanced and modified RT-PCR assays that may also provide better detection sensitivity than 10^2^ CFU/ml, through modifications to the DNA extraction procedure and PCR conditions. However, our developed immunoliposome-based immunomagnetic concentration and separation assay provides practical applicability due to overcoming the limitation of a PCR assay that employs several critical steps that require expert handling and incur higher costs.

### Cross-reactivity

**Figure 8:**
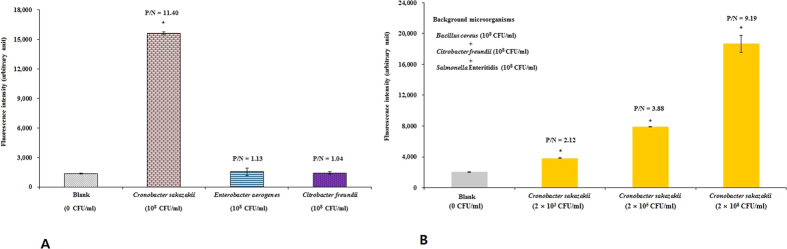
Cross-reactivity of the developed immunoliposome-based immunomagnetic concentration and separation assay with other genera of foodborne pathogens spiked into powdered infant formula (**A**) and effect of background microflora in powdered infant formula on the detection of *Cronobacter sakazakii* using the developed immunoliposome-based immunomagnetic concentration and separation assay (**B**). All experiments were conducted three times, and data represent the mean ± standard deviation. The coefficient of variation for fluorescence intensity (n = 6) was below 15%. *Significantly different (*P* < 0.05) compared with blank.

To further confirm the specificity and practical application of the developed immunoliposome-based immunomagnetic concentration and separation assay, the cross-reactivity of the assay was checked using foodborne pathogens belonging to different genera (Fig. 8A). The developed assay method showed a lack of significant cross-reactivity in PIF samples contaminated by bacterial species belonging to other genera, even other *Cronobacter* species, as previously reported by our research group^15^. As bacterial species possess various antigen-binding sites on their surface, which can interfere with binding of the target pathogen to the antibody in contaminated food samples during the development of immunoassays, it is very important to cross-check the specificity of the developed immunoassay in a food matrix^44^. In addition, we confirmed the specificity of the developed method in a pure bacterial culture, as only the specific antigen-antibody interaction was detectable^15^. In another study, we developed an immunoliposome-based fluorescence assay that also showed no cross-reactivity with any other test pathogens except for the target pathogen *C. muytjensii*^46^. The difference in recognition between the targeted bacteria (positive value; P value) and non-targeted bacteria (negative value; N value) is an important parameter determining the effectiveness of an immunoassay^47^. In the present study, non-targeted bacteria showed P/N values of 1.13 and 1.04 (<2), which were considered a negative result, whereas targeted bacteria (*C. sakazakii*) showed a P/N value of 11.40 (>2), which was considered a positive result (Fig. 8A).

### Effect of background microflora

Specific criteria are applied to the analysis of food samples; one key aspect to consider is that the target microorganisms must be detected amidst background microflora, whose composition will vary greatly depending on the type of food sample. Moreover, food matrices vary and can substantially influence the analytical process. Routine laboratories must handle a large number of samples, and costs play a more important role in clinical samples. In addition, background microflora can cause interference during pathogen detection. Therefore, to visualize the effects of background microflora, the relative detection sensitivity of the developed assay was determined using spiked PIF samples with different concentrations of *C. sakazakii* cells (2 × 10^3^, 2 × 10^5^, and 2 × 10^8^ CFU/ml) as well as a high background cell concentration (10^8^ CFU/ml) of other bacterial strains, including *B. cereus*, *C. freundii*, and *S.* Enteritidis. In the present study, *B. cereus* and *S.* Enteritidis were selected in light of a report from the World Health Organization on the high likelihood of PIF contamination by *B. cereus* and *S.* Enteritidis^48^,^49^. Giammanco *et al.*^49^ also reported that *Salmonella* species and *Cronobacter* species (formerly *Enterobacter sakazakii*) are common microorganisms that contaminate PIF. Hence, determining the effects of these microbes in PIF is important. Giammanco *et al.*^49^ reported that *C. freundii* isolated from PIF was misidentified as *Cronobacter* species in a molecular epidemiological survey. The phylogenetic tree also shows that *C. freundii* and *Cronobacter* species are very closely related based on their small Mahalanobis distance compared with other species. Hence, researchers suggest that *C. freundii* may also be an under-reported cause of bacterial infection, especially in high-risk neonates, due to misidentification.

It was observed that the background contaminant microflora had no effect on the applicability, detection sensitivity, or efficiency of the developed assay. As demonstrated in Fig. 8B, even a lower spiked level of *C. sakazakii* and higher cell numbers of other background contaminants had no effects on detection sensitivity, confirming no interference from the background microflora or the matrix. This result was likely due to the use of immunomagnetic nanoparticles to remove any possible interference in the PIF samples. Although the current developed method has only been used for the detection of *C. sakazakii* in PIF samples, it could also be suitable for detecting *C. sakazakii* in other food samples. However, our method is limited due to its lack of multiplexing capability, which may require further work to overcome and to validate the practical and industrial usefulness of the method. To overcome this problem, our next goal is to develop a new strategy for multiplexing detection of *C. sakazakii* using our developed assay with 96-well microtiter plates positioned into magnetic plate separators. This work may further improve the detection sensitivity in addition to providing a multiplex platform. Alternatively, the developed method represents an effective strategy for the detection of *C. sakazakii* due to the following two aspects. First, the IgG coated on the magnetic nanoparticles only captures target bacteria, supporting the efficiency and practical applicability of the developed assay in food samples. Another notable aspect is the potential application of a similar or improved strategy using the developed concept for detecting other foodborne pathogenic bacteria by selecting and developing different antibodies.

## Conclusions

In conclusion, we developed a rapid fluorescence detection system by incorporating target antibody into liposomes and magnetic nanoparticles. The data obtained in this study demonstrate that a food trial-based optimized assay could be successfully utilized for sensitive and rapid detection of *C. sakazakii* in PIF samples. The detection limits of *C. sakazakii* in spiked PIF samples with 6 h of pre-enrichment was 2 cells/10 g of PIF. Moreover, the developed assay was able to detect 3.8 × 10^3^ CFU/ml of *C. sakazakii* in PIF samples without any pre-enrichment. Future directions of research include generalizing this method for the detection of all species of *Cronobacter* by constructing a multiplexing detection format.

## Additional Information

**How to cite this article**: Shukla, S. *et al.* Detection of *Cronobacter sakazakii* in powdered infant formula using an immunoliposome-based immunomagnetic concentration and separation assay. *Sci. Rep.* **6**, 34721; doi: 10.1038/srep34721 (2016).

## Supplementary Material

Supplementary Information

## Figures and Tables

**Table 1:** List of commercial kits available for the detection of *Cronobacter* species.

Detection kits	Target pathogens	Principle of detection	Company
TaqMan *Cronobacter* detection kit	*Cronobacter sakazakii*	DNA-based PCR	Applied Biosystems
VIT *Cronobacter* species/*Enterobacter sakazakii* detection kit	*Enterobacter sakazakii (Cronobacter* species)	VIT^Ⓡ^ gene probe technology (PCR-based)	Vermicon Solutions
Foodproof ^Ⓡ^ rapid detection kit	*Enterobacter sakazakii (Cronobacter* species)	PCR-based	Biotecon Diagnostics
BAX^Ⓡ^ system PCR assay	*Enterobacter sakazakii (Cronobacter* species)	PCR-based	Dupont Qualicon BA^Ⓡ^ System
Assurance GDS^TM^	*Enterobacter sakazakii (Cronobacter* species)	PCR-based	Biocontrol GDS Rotor Gene

**Table 2:** Particle size, zeta potential, and polydispersity index of liposomes, magnetic nanoparticles and immunomagnetic nanoparticles.

Type of nanoparticle	Size	Zeta potential	Polydispersity index
Liposome particles	244.3 nm	−42 ± 7.72 mV	0.246
Magnetic nanoparticles	163.9 nm	−53 ± 9.89 mV	0.289
Anti-*Cronobacter sakazakii* immunoglobulin G-conjugated magnetic nanoparticles (immunomagnetic nanoparticles)	373.4 nm	−46 ± 4.37 mV	0.266

**Table 3:** Efficiency of anti-*Cronobacter sakazakii* immunoglobulin G conjugation with magnetic nanoparticles.

Antibody	Amount of antibody	Coefficient of variation
Initial antibody	8.35 ± 0.00 mg/ml	—
Free antibody	0.96 ± 0.01 mg/ml	1.88%
Bound antibody	7.39 ± 0.59 mg/ml	1.36%
Conjugation efficiency	88.5%	2.62%

Conjugation efficiency (%) = Bound (initial–free) antibody/initial antibody × 100. The antibody concentration was measured with the Bradford assay. All experiments were conducted three times, and data represent the mean ± standard deviation. The coefficient of variation (%) was determined via intra assay test by running 10 replicates of the experiment in one analytical run.
