# Supplementary material for: Detection of Cronobacter sakazakii in powdered infant formula using an immunoliposome-based immunomagnetic concentration and separation assay
Source: Sci Rep. 2016 Oct 10;6:34721. doi: 10.1038/srep34721 (PMC5056387; doi:10.1038/srep34721)
Supplement: Supplementary Information [file srep34721-s1.doc]

**Supplementary Data**

**Detection of *Cronobacter sakazakii* in powdered infant formula using an immunoliposome-based immunomagnetic concentration and separation assay**

**Shruti Shukla^1§^, Gibaek Lee^1§^, Xinjie Song^1^, Jung Hyun Park^1^, Hyun Jung Cho^2^, Eun Ju Lee^3^, & Myunghee Kim^1^,***

^1^Department of Food Science and Technology, Yeungnam University, 280 Daehak-ro, Gyeongsan-si, Gyeongsangbuk-do 38541, Republic of Korea

^2^Division of Safety Analysis, National Agricultural Products Quality Management Service, Gimcheon-si 39660, Republic of Korea

^3^Department of Medical Biotechnology, Yeungnam University, 280 Daehak-ro, Gyeongsan-si, Gyeongsangbuk-do 38541, Republic of Korea

**^§^Both authors contributed equally.**

**Running title:** Nanoparticle-based immunoassay for *Cronobacter* *sakazakii* detection in powdered infant formula

To whom correspondence should be addressed:

**Prof. Myunghee Kim***

Department of Food Science and Technology, Yeungnam University, 280 Daehak-ro, Gyeongsan-si, Gyeongsangbuk-do 38541, Republic of Korea

Phone: +82-53-810-2958; Fax: +82-53-810-4662; E-mail: [foodtech@ynu.ac.kr](mailto:foodtech@ynu.ac.kr)

**Results**


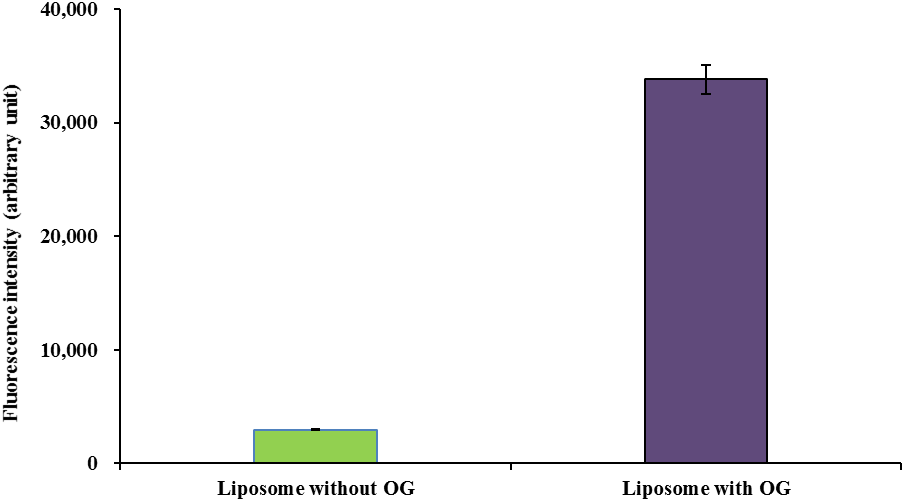


**Figure S1.** Integrity measurement of sulforhodamine B-tagged liposomes. Values represent the mean of three measurements. Error bars represent the mean ± standard deviation. The coefficient of variation for fluorescence intensity (n = 6) was below 15%.
